# The shifting of traditional understanding of citizenship due to international migration

**DOI:** 10.3389/fsoc.2025.1568332

**Published:** 2025-05-21

**Authors:** Angela Paparusso, Catherine Wihtol de Wenden

**Affiliations:** ^1^Institute for Research on Population and Social Policies of the National Research Council (CNR-IRPPS), Rome, Italy; ^2^Centre National de la Recherche Scientifique (CNRS), Sciences Po, Centre de Recherces Internationales (CERI), Paris, France

**Keywords:** citizenship, nationality, migration, globalization, transnationalism, policies, European Union, France

## Abstract

This article analyzes how migration has profoundly influenced the conception of citizenship by challenging the traditional state-citizen relationship and introducing transnational and European citizenship, which disconnects nationality from civic rights. In particular, our research questions are how migration has reshaped the conception of citizenship and what challenges migrants, refugees, stateless individuals, asylum seekers and environmentally displaced persons face in obtaining legal status in host countries. France is presented as a case study of the negotiations shaping the evolution of the conception of citizenship in relation to migration. Our literature review-based research highlighted the dissociation between nationality and citizenship and the emergence of migrant practices, like dual citizenship, transnationalism, and naturalization, which have redefined borders and forms of belonging, focused on residency and participation. Countries with long immigration histories have embraced diversity, multiculturalism, and anti-discrimination policies to integrate migrants. However, most people from the Global South face barriers to legal migration, resulting in populations without formal status, amid rising resistance to greater inclusion led by populist forces.

## 1 Introduction

Over the past 30 years, the world has become increasingly mobile, driven by the fall of the Iron Curtain in former communist regimes, including China, and in many Southern countries that began granting access to passports. This shift marked the emergence of the right to emigrate as a universal right, although borders have progressively tightened against immigration, leaving many people excluded from the access to citizenship (Wihtol de Wenden, 2017).

Globalization is the main driver of various forms of mobility, which has reshaped the concept of citizenship, traditionally governed by Nation States. However, the emerging gap between the universal right to emigrate and the discretionary right to immigrate is creating a new form of disorder. Mobility is considered as a factor of human development that characterizes contemporary age, as highlighted by the United Nations Department of Economic and Social Affairs (UNDESA), Global Forum on Migration and Development (GFMD) and the 2018 Marrakech Global Compact. However, the unequal access to the right to move globally is leading to the emergence of different forms of citizenship, many of which are negotiated, thus resulting in distinct forms of agency.

Referring back to the definition of “universal citizenship” of Immanuel Kant (in “Project for a Perpetual Peace: A Philosophical Essay” of 1796) (Kant, 2021), which grants all individuals the right to move across the globe, and to the idea of “liquid modernity” of Bauman (2000), which frames mobility as a characteristic of both citizens and various forms of exchange – trade, knowledge, finance, and information – one finds a paradox. Those who are sedentary often possess more rights than those who are mobile. This issue is particularly evident in international migration, where many migrants find themselves without legal status or any recognized form of belonging.

The aim of this paper is to analyze how migration has profoundly changed the conception of citizenship by challenging the traditional state-citizen relationship and introducing transnational and European citizenship, which disconnects nationality from civic rights. Our research questions are how migration has reshaped the conception of citizenship and what challenges migrants, refugees, stateless individuals, asylum seekers and environmentally displaced persons face in obtaining legal status in host countries. We will answer these research questions by reviewing the international scientific literature on the topic.

After this Introduction, the second section of the paper will analyze how migration has disrupted traditional conceptions of citizenship, reshaping the relationship between the State and its citizens. The third section will explore the different ways in which citizenship is negotiated, including dual citizenship, asylum and irregular migration. The forth section will focus on France as a case study of the negotiations surrounding the evolution of citizenship in response to international migration. Finally, the last section concludes the paper.

## 2 Citizenship challenged by migration

### 2.1 The globalization of migration

This section aims to examine the impact of migration on traditional notions of citizenship and explore how both migrants and host countries have adjusted to these changes over time.

Migration is a structural phenomenon, rooted in migration systems of interdependency. The concept of migration systems, initially developed by Massey (2008) in the context of the United States-Mexico region, highlights the interconnected nature of migratory flows. Most migration systems are shaped by a combination of disparities – demographic, economic, cultural, and political (e.g., democracy vs. authoritarian regimes). These disparities persist in countries of origin, leading many individuals to see migration as the only viable solution (Carling, 2024). In this regard, Sassen (2009) discusses the concept of “global city”, which marginalizes a portion of the population, pushing it to the peripheries or into migration.

In the early twenty-first century, migration has become a global phenomenon. This is not solely due to the scale of migration flows – 284 million international migrants, representing 3.7% of the global population, according to the last estimates of the Department of Economic and Social Affairs, Population Division of the United Nations – but also because of its reach. No region or country is untouched by migration; all are engaged in emigration, immigration, or transit flows, and often in all three simultaneously.

Migration flows have become increasingly diverse, encompassing a wide range of groups: refugees and asylum seekers, women (who now account for half of global migration flows), children (including a growing number of unaccompanied minors), and highly skilled migrants. In particular, family migrants often exceed the number of workers entering traditional immigration countries. Additionally, many individuals lack a defined status, such as undocumented migrants – approximately 12 million in the U.S., 5 million in Europe, according to the last estimates of the Pew Research Center, and countless others in the Global South.

The number of migrants moving to the South has now matched the number heading to the North. When considering South-South migration, toward emerging countries and Gulf States, as well as environmentally displaced persons migrating from South to North, the numbers become even more balanced. Additionally, skilled migrants from the North to the South, retirees seeking warmer climates in the South, entrepreneurial ventures by second and third generations in their parents' countries, and the exploitation of raw materials by Northern countries in the South, all contribute to a migration flow that equals the numbers migrating to the North. This includes both South-North migrants (refugees and workers) and North-North migrants (skilled individuals, students with exchange programs, and tourists who choose to settle).

The distinctions between categories of migrants have become increasingly blurred, as many individuals seeking family reunification or asylum are also in search of employment, often sharing similar sociological profiles (Ambrosini, 2020). Many individuals attempt to cross borders as asylum seekers because other legal pathways for entry are closed. This results in mixed categories of migrants, who may resemble refugees or workers, although the distinction between these two groups is often challenging to define clearly (Ambrosetti and Paparusso, 2018). This contrasts sharply with the Cold War era, when political dissenters had profiles that were markedly different from those of migrants during periods of economic growth. Today, a migrant entering a country often has a higher level of qualification than the average population in the destination country and is significantly more productive than in the country of origin (World Bank, 2023). If qualified, an individual may move through various statuses, ranging from undocumented to legal, following a process of regularization based on his/her skills (Ambrosini, 2018).

While two-thirds of the global population lacks the right to move freely beyond their national borders, transnational connections persist. These include family networks abroad, advancements in communication technologies, and the role of remittances. Furthermore, members of second or third generations from long-established migrant families often hold dual citizenship, enabling them to cross borders more easily. This facilitates a lifestyle that blends settlement and mobility, allowing individuals to move in and out of countries with greater freedom (de Haas and Fokkema, 2011).

### 2.2 What does citizenship mean in a world on the move?

Although the typical framework of the national order envisions citizens living in the state where they hold citizenship and residency, bound by the rights and duties of that state, this traditional understanding has shifted with increasingly mobile populations. However, many migrants lack recognition in a world characterized by mass migration. Many categories of migrants, such as undocumented individuals, rejected asylum seekers, stateless people or environmentally displaced persons do not find a proper legal recognition. In this regard, some advocate for defining mobility as a global public good and a human right for the twenty-first century, while others argue that mobility disrupts the established order of Nation States (Geddes, 2021).

A new gap has emerged between the universal right to emigrate, as stated in Article 13 of the Universal Declaration of Human Rights of 1948, and the restrictive, often discretionary right to immigrate, creating a new paradox between the right to emigration and the right to immigration. Since the 1990s, the widespread access to passports has opened borders to many migrants. The inequality of the right to move across the world mostly depends on national passports and visa regimes, which are built on the notion of “migration risk”. This context gives rise to various forms of mobility – both legal and illegal – and residence statuses that are often negotiated, while also fostering different forms of exclusion.

With migration, citizenship can no longer be understood in its traditional sense (Castles, 1997; Castles and Davidson, 2000). Migrants are often viewed as future citizens of their host countries, while also maintaining ties with their country of origin (Finotelli et al., 2025). However, many migrants have become second-class citizens (*denizens*) in Northern Europe, gaining local political rights without full citizenship (Hammar, 1990). Globalization has introduced a segmented and often hierarchical form of citizenship, even if the space of expression of citizenship has expanded at local level and dual citizens can experience citizenship in both their countries of origin and destination.

The second generation has further complicated the concept, by introducing ideas of belonging, multiple allegiances, and transnational citizenship through networks. Sayad's (1999) concept of the migrant worker's “double absence” – being a non-citizen in both the country of origin and the country of arrival – has evolved into a “double presence”, where many migrants now live “here and there” (Crawley and Jones, 2021). Pendular migrants, who embrace mobility as a way of life, can do so when they benefit from flexible visa and circulation options. New local citizens and non-European migrants exemplify a form of citizenship based primarily on residence and participation, detaching citizenship from migration, and introducing new values into the traditional understanding of citizenship, such as anti-discrimination and diversity.

### 2.3 Dissociation between nationality and citizenship in Europe, and *jus soli vs. jus sanguinis*

The dissociation between citizenship and nationality, introduced by European citizenship under the Maastricht Treaty of 1992, as well as by European countries granting local citizenship to non-nationals, represents one of the most significant impacts of European integration on these concepts (Wihtol de Wenden, 1997). Since the mid-1970s, northern European countries have paved the way for this shift by extending local political rights to all foreigners. For instance, Sweden introduced such measures in 1975, followed by Denmark in 1981, the Netherlands in 1985, and Belgium in 2000, followed by certain regions in Switzerland, such as Neuchâtel and Jura. Additionally, Great Britain granted these rights to Commonwealth citizens. Today, 15 of the 27 European Union (EU) member states (post-Brexit) have granted local citizenship to non-European nationals. Local citizenship has been defined by Bauböck (2006, p. 24) as a “residential citizenship that is disconnected by the nation-state membership”.

EU citizens residing in a member state other than their country of origin acquire local citizenship in the host country without becoming nationals of that state, if they are engaged in local political life as voters or candidates. Therefore, citizenship in the EU resembles a series of concentric circles. At the center are national citizens who reside in their country of nationality. Surrounding them are EU citizens, followed by non-European long-term residents, non-European short-term residents, refugees, asylum seekers, and undocumented migrants. Those in the outermost circles are deprived of political expression, even though they live within a democratic space (Wihtol de Wenden, 1997).

These new forms of citizenship, detached from nationality, emphasize residence, local ties, grassroots participation, and multiple identities that embrace diversity. During the 1990s, most EU member states engaged in debates over reforms to nationality laws, shifting toward greater inclusivity. Many countries, such as Belgium, France, Portugal and Spain, adopted *jus soli* (right of the soil) alongside *jus sanguinis* (right of blood), balancing both principles to facilitate citizenship access for newcomers and their children born in the host country (Paparusso, 2019). Notable exceptions include Italy, where Italian-born foreign nationals under the age of 18 can apply for citizenship within 1 year of turning 18 (Law n. 91/1992). Conversely, Germany introduced *jus soli* for those born in Germany, if at least one parent has a permanent residence permit and has been residing in Germany for at least 8 years (German Nationality Act of 1999, which entered into force in 2000) (idem).

### 2.4 Transnational citizenship and multiculturalism

The concept of transnationalism, theorized by Schiller et al. (1992), examines the interplay between states, networks, and non-state actors. According to Bauböck (1994), transnational citizenship is rooted in consensual belonging and voluntary participation, reflecting Hirschman's model of exit and loyalty (Hirschman, 1970). This framework challenges the traditional bond between citizens and the state, as the right to mobility often contradicts the fixed relationship inherent in the classical concept of citizenship.

Transnational migration gives rise to forms of transnational citizenship characterized by multiple allegiances, political influence, and sometimes the involvement of countries of origin in the political affairs of countries of destination. In some cases, countries of origin leverage migration as a tool of diplomacy. Diasporic states, such as Turkey or Morocco in Europe, strengthen their influence by utilizing migration networks established by their citizens settled across various EU nations.

Transnational citizenship, as studied by Bauböck (1994) and Soysal (1994), shows how far citizenship can be experienced and mobilized through migration. This form of citizenship transcends national borders, expanding the limits of the Nation State and positioning transnational citizenship as an alternative to classical definitions of citizenship.

Immigration countries have historically questioned their ability to assimilate newcomers, leading to the emergence of multiculturalism as a negotiated approach to citizenship and national identity. Multicultural citizenship has gained legitimacy in Europe (recognized as “diversity” in the Lisbon Treaty of 2007), as well as in Australia and Canada, where it is enshrined in constitutional frameworks (Elias et al., 2021). In the United States, multiculturalism is framed around universal values such as non-discrimination, cultural pluralism and interfaith dialogue (Alba, 1999).

However, multiculturalism faces significant opposition today, particularly from far-right parties (Joppke, 2021). Also the academic literature (Duyvendak and Scholten, 2011; Kymlicka, 2012; Koopmans, 2010) has shed light on the debate about the effectiveness of multiculturalism in fostering migrants' socio-political integration. In particular, it has been argued that since it emphasizes ethnic and cultural particularisms, multiculturalism can risk of “reinforcing ethnic stratification and ethno-cultural conflict” (Bosswick and Heckmann, 2006, p. 5), producing isolation and discouraging the process of reducing gaps between nationals and non-nationals. Moreover, multiculturalism often clashes with the myth of national homogeneity, which may foster artificial internal borders, based on ethnicity or religion, and a deep suspicion of the “other”. Nevertheless, some scholars have concluded that multiculturalism does not undermine migrants' integration (Wright and Bloemraad, 2012).

### 2.5 The transnational social question

Many forms of transnational citizenship have emerged, characterized by various expressions of dual presence at both infra- and supra-national levels, driven by sociological practices that transcend borders, such as economic entrepreneurship, mixed marriages, migrant family networks, associative life and religious diaspora. More broadly, mobility implies the definition of the rights of mobile citizens, which in turn weakens the traditional relationship between the citizen and the State.

Faist (2004, 2018) examined the complexity of migration through a transnational lens, focusing on its intersection with social questions. His approach emphasizes social protection within a global framework, highlighting the lack of a global migration governance that currently seeks to reduce inequalities between states. Efforts are limited to addressing disparities within countries despite significant variations in social protection systems. The absence of a global migration regime means that migration governance does not address international social inequalities or heterogeneities. The primary objective of global migration governance – understood as the collection of norms that guide states and stakeholders in addressing migration – should be to mitigate the adverse impacts of restrictive policies on human mobility while promoting global stability. Conversely, restrictive policies often intensify social exclusion, denying certain groups access to social protection, leading to tragic loss of life at borders, and fueling hostility and resentment toward migrants in host countries, ultimately affecting their overall wellbeing (Paparusso, 2021).

Transnational migration, facilitated by cross-border networks, gives rise to forms of transnational citizenship characterized by multiple allegiances and new challenges influencing citizenship, alongside diverse practices of solidarity. Faist (2004) explores these dynamics by presenting two key ideas to examine the social implications of migration. (1) The traditional model of internal class struggle is no longer relevant; instead, social and cultural inequalities now exist between the Global South and the Global North. (2) The place of birth and current residence have become the primary sources of global inequality and heterogeneity.

In recent years, heterogeneities – ethnic, religious, linguistic – have intensified in the context of immigration and emigration, particularly along the North-South divide. These dynamics are transnational, shaped by migrants' cross-border connections, remittances, corporate recruitment of workers, and the transnationalization of lifestyles. However, economic and political inequalities between countries now surpass those within nation-states. Additionally, the rural-urban divide represents another critical axis of disparity (Triandafyllidou et al., 2024).

We are witnessing a transition from class-based differences to location-based disparities, where citizenship is increasingly tied to geographic location and regulated through mobility rights and visa systems. Citizenship, rather than being solely determined by one's social class, is now heavily influenced by where one is born or where they hold legal status. This has created a system where people from certain countries – often those in the Global South – face significant barriers to migration and access to basic rights due to the limitations of visa policies or restrictive immigration laws in wealthier nations (Alberti and Sacchetto, 2024). Countries unable to address inequality, poverty and violence become the source of refugees, where “exit” often replaces “voice” as a response. Many migrants seek social protection and contribute through remittances and the exchange of information for care and health services. In these regions, political mobilization and transnational movements emerge, advocating for justice and combating social inequalities (idem).

Migration underscores the critical significance of place – both residence and belonging – in shaping opportunities, whether seen as chance or fate, particularly in countries of origin. Paradoxically, at a time when geographic location outweighs indicators like the Human Development Index (HDI) in determining life chances, opportunities for international migration are increasingly constrained by inequalities in migration rights (such as access to visas) and stricter border controls. In response, a grassroots transnationalism is emerging, revealing the unequal right to migrate as a key driver of stratified inequalities. This dynamic results in selective mobility for a privileged few and widespread immobility for the majority. Consequently, the transnational social question is now simultaneously a global and local issue (Faist, 2009).

To conclude this section, with increasing mobility and the dual affiliations of settled individuals, new forms of transnational citizenship are emerging. These forms incorporate plural allegiances and policies in both host and origin countries, which establish connections with their members by practicing a diplomacy of migration. This includes support for associative life, oversight of religious networks, facilitation of é*lite* networks, recognition of dual citizenship, special incentives for investment in countries of origin, and granting voting rights in these countries. Meanwhile, second-generation migrants reconstruct their identities, while others may find themselves entirely excluded (Wihtol de Wenden, 2015).

## 3 Multiple forms of negotiated citizenship

While the norm of the national order assumes a citizen residing in the state where he/she holds citizenship and is subject to its rights and duties, the dynamics of belonging and allegiance have shifted with the rise of mobile populations. This is especially true for individuals for whom the Nation State no longer functions, such as refused asylum seekers, stateless individuals, and undocumented migrants. In other cases, the concepts of allegiance and belonging lose their significance in countries where one can essentially “buy” citizenship through investments, such as purchasing a large property, establishing a business, or acquiring a so-called “golden passport” (Wihtol de Wenden, 2017).

Within these considerations, this section is devoted to shed light on various forms of agency that shape the evolution of citizenship, such as dual citizenship, irregular migration, asylum, and irredentism. Agency can be understood as the capacity of individuals to act independently and make their own choices within social structures. Using various forms of activism and militancy, migrants develop multiple forms of negotiated citizenship. Similarly, activist non-governmental organizations (NGOs) play a crucial role in advocating for greater recognition of migrants' rights.

Dual citizenship facilitates the experience of a double presence in the country of origin and in the country of destination. Although some countries of origin prohibit dual citizenship, this practice is becoming less common. The opportunity to acquire dual citizenship is supported by the persistence of *jus sanguinis* in many Islamic and Asian countries and the extension of *jus soli* in immigration countries, especially in the EU, as highlighted in the previous section. Historically, migrants who acquired the nationality of their host countries before dual nationality was widely accepted, were often viewed as unfaithful citizens by their countries of origin (Wihtol de Wenden, 2006). However, over time, many emigration countries began to recognize the advantages of having dual citizens. In immigration countries, the nationality of origin often became a “dormant nationality” for migrants who naturalized. During the Cold War, refugees, in particular, frequently saw no hope of returning to their countries of origin. However, with the resolution of conflicts, many have been able to return, albeit often losing their refugee status under the “cessation clause” of the Geneva Convention. Today, dual citizens view themselves as ordinary citizens in both their countries of origin and residence. The possibility that naturalized citizens can retain their native citizenship does not change their sense of belonging to their country of origin (Howard, 2005). However, they are sometimes perceived as potential security risks, particularly in the context of terrorist threats, or as individuals with divided loyalties. Notwithstanding this perception, they often serve without any sense of dual allegiance in military conflicts, such as those in Afghanistan, Iraq, or the Sahel. Dual citizenship can also help to navigate restrictive travel regimes, especially for those who lack access to passports from countries like those in Europe, the United States, or Canada.

Irregular migration consists of individuals who are denied any form of protection or integration into the host country and it includes undocumented migrants, rejected asylum seekers, individuals with serious illnesses who lack protection, and unaccompanied minors who have reached the age of majority. Irregular migrants challenge the traditional conception of citizenship since they move despite the restrictions that immigration countries impose on their movement (Ambrosini and Hajer, 2023). However, although often portrayed as passive victims, irregular migrants exhibit significant agency in advocating for legalization and for being incorporated into the societies in which they reside despite their irregular status (idem). They are generally supported by human rights organizations and their access to rights or legal status often depends on negotiations with public authorities, who hold discretionary powers to regularize their status (Gschwind et al., 2025).

Another way in which migrants exercise agency is through advocacy for refugee rights. During the Cold War, acceptance rates were quite high, as asylum seekers were often viewed as victims of communist regimes. Today's asylum seekers are collective groups persecuted not only by their states of origin but also by civil society, due to their ethnic, religious, or sexual identities. This evolving refugee profile challenges traditional notions of citizenship and territorial belonging. Refugees, as international actors, exist within an international society of sovereign states as exceptions to the norm. Their lack of state representation is perceived as a disruption to the international order. The 2015 migration crisis highlighted the significance of political asylum for those fleeing wars and conflicts, as legal access to EU states was almost impossible for individuals seeking work. Asylum thus became a pathway to obtain legal status for those whose narratives of persecution were accepted by host states. Many migrants are both fleeing unstable countries – sometimes engulfed in civil wars – and seeking economic opportunities. However, asylum is often the only legal option available for individuals without documentation, especially in the absence of open channels for economic migration (Wihtol de Wenden, 2017).

According to the last estimates of the United Nations High Commissioner for Refugees (UNHCR), 4.4 million stateless people live around the world, despite efforts by the 1954 UN International Convention on Statelessness to reduce such cases. Many of them reside in Bangladesh, including the Rohingyas from Myanmar, who are denied legal recognition in their home country. Myanmar's Constitution defines its population based on a list of ethnic groups, deliberately excluding the Rohingyas. As a result, many Rohingyas seek refuge in neighboring countries, primarily Bangladesh, which does not grant them official status. Stateless populations are also found in other regions, such as the Great Lakes region in Africa. In Europe, the Baltic States did not grant citizenship to Russian settlers who failed language proficiency tests in Baltic languages. These individuals hold so-called “gray passports”, which allow them to travel to Russia without a visa but provide limited access to EU countries, mostly neighboring the Baltic region. Unlike refugees, who retain their former citizenship, stateless individuals have no citizenship and thus lack diplomatic protection. Some stateless people are also victims of denationalization policies enacted by certain governments. While there is a global trend toward reducing statelessness, many countries continue to overlook or neglect the plight of stateless populations (Brunborg, 2024).

Environmentally displaced people, often referred to as “climate refugees”, lack any formal legal status. Several factors explain why international actors have delayed recognizing their status. First, UNHCR does not consider them refugees under the Geneva Convention. Second, defining and quantifying environmentally displaced people is challenging. Most of them are internally displaced within the Global South or migrate to other Southern countries, making precise data scarce. This lack of clear definitions affects their recognition, leading to limited statistical data and minimal acknowledgment of their plight. The Global Migration Data Analysis Center (GMDAC) of the International Organization for Migration (IOM) estimated that there were 26 million people displaced by environmental factors in 2023; they are projected to reach 216 million by 2050 according to the World Bank. Third, reports from the Intergovernmental Panel on Climate Change (IPCC) have categorized most climate-related migrations as regional. This has led to proposals for regional statuses, which raise concerns for instance about the issue of comparing the situation of people from the Halligen Islands in the North Sea (Germany and Denmark) to those from regions like the Sahel or Bangladesh, among the poorest in the world. Fourth, the causes of environmental displacement, such as droughts or conflicts exacerbated by climate change, complicate the evaluation of victims, further hindering solutions. Nonetheless, some progress has been made through negotiations. In the Pacific, for example, Tuvaluans have been temporarily welcomed in Australia and New Zealand, and proposals like climate visas have been introduced. Additionally, legal proceedings have been used to hold states accountable for failing to address the conditions forcing displacement. However, despite initiatives such as the 2011 Nansen Initiative in Geneva, no comprehensive solution has been adopted so far. Efforts by countries like Bangladesh at United Nations conferences, the inclusion of this issue in the 2018 Marrakech Global Compact, and discussions at GFMD since 2010 have yet to result in concrete measures (Apap and Harju, 2023).

Finally, irredentism refers to the practice of granting citizenship to individuals who are not formal citizens of a country but have ancestral ties. This phenomenon challenges the traditional state-citizen relationship, which is typically based on territorial boundaries. Historically, after the First and Second World Wars, many populations lost their citizenship and lacked the right or the desire to acquire citizenship in their new countries. Today, one notable example of irredentism is Hungary. After the First World War, Hungary lost two-thirds of its territory. Following the fall of the Iron Curtain, Hungarian citizenship became accessible to Romanian populations of Hungarian descent who continued to live in Romania. This issue has become more prominent in Ukraine, where obtaining a Hungarian passport provides access to the EU citizenship, allowing individuals to travel, settle, and work visa-free across the EU. In the southwest of Ukraine, formerly part of the Austro-Hungarian Empire, as well as in Czechoslovakia, Moldova, and Romania, approximately 1.5 million people have acquired Hungarian passports. This trend aligns with Hungary's nationalist policies and its efforts to address its declining population (Brie and Polgar, 2011).

## 4 France as a case study of the negotiations surrounding the evolution of citizenship

As stressed by Simon (2012), although multiculturalism in France is still rather rejected by both the political and public debate, France is a multicultural country, since its population is largely and increasingly diverse. Minority identities are not necessarily conflicting with the sense of attachment to France: they can be integrated into the host society without renouncing to their identity. Moreover, international migration and the European integration process have redefined the conception of citizenship, distinguishing legal status, civic identity, and civic practice. The European integration process, in particular, has introduced a hierarchy of membership between Europeans and non-Europeans, while transnational citizenship has emerged, encompassing diasporic political belonging across both origin and host countries. For these reasons, we believe that the case of France is illustrative of the evolution of the understanding of citizenship due to international migration. To present this case study, we primarily refer to a previous work by Wihtol de Wenden (2006).

France is considered the birthplace of modern citizenship, shaped by the Revolution and marked by a shift from *jus soli* to *jus sanguinis*, introduced by Napoleon Bonaparte in the Civil Code of 1804. This change was inspired by Enlightenment ideas and implemented in the countries conquered by Napoleon. However, today, France is grappling with the resurgence of far-right rhetoric advocating for the abolition of *jus soli*.

The distinction between citizenship and nationality emerged with the French Revolution. Citizenship preceded nationality, as at the time, nationality was not a major concern; most people did not migrate, and there was no strong sense of belonging to a nation. The revolutionary citizen of 1789 was, above all, a man who embraced the ideals of the Revolution – freedom, equality of rights, the right to property – and who actively participated in assemblies and political clubs. This concept drew inspiration from Greek democracy and the Roman Republic: the citizen was envisioned as someone entirely devoted to public values, “a hero of wisdom and probity” (Saint-Just). Citizenship reflected the principles of the Enlightenment: the social contract (Rousseau), freedom of conscience (Voltaire), and the separation of powers among the executive, legislative, and judiciary branches (Montesquieu). It held a philosophical dimension, as defined in the 1789 Declaration of the Rights of Man and of the Citizen (*Déclaration des droits de l'homme et du citoyen*).

### 4.1 Nationals but not citizens

During the French Revolution, most of the population lived in the territory of birth. It was not necessary to be a national to become a citizen; participation held greater importance than nationality. Some foreigners, such as Anacharsis Cloots and Thomas Paine, were active members of the assemblies (*Constituante*) and were therefore granted the “status” of citizen. The Constitution of 1793 even recognized the granting of citizenship for civic services rendered to the state, such as feeding and caring for a child or helping an elderly person. This emphasis on civic values was later echoed during the Paris Commune of 1871, when some foreigners were granted citizenship. The idea that one could be a citizen without being a national resurfaced much time later, during the political campaigns of the 1980s. It was used as an argument to advocate for granting local political rights to foreigners and expanding their access to new rights.

Conversely, there have been many historical instances where nationals were not considered citizens. For example, women were excluded from citizenship until 1944, and young people were not granted full citizenship rights until the voting age was lowered from 21 to 18 in 1974. Members of the military were deprived of voting rights during the Third Republic and referred to as the “big dumb” (*la grande muette*). Similarly, certain disabled individuals, such as those deemed insane, and individuals stripped of their civic rights by judicial decision (*déchéance des droits civiques*), were also excluded. Under colonial rule, indigenous populations were denied access to citizenship, and a hierarchy of citizenship existed, determined by the territory of birth and level of education. In Algeria, then a French colony, full access to citizenship was granted only in 1947, while Jews of Algerian origin, primarily of Spanish descent, were granted French citizenship much earlier, at the end of the nineteenth century, under the Crémieux Decree (*Loi Crémieux*) of 1870.

Paradoxically, despite its revolutionary roots, citizenship has only recently become a central theme in France. During the “Glorious Thirty” years of 1945–1975 (*Les Trente Glorieuses*), citizenship was rarely discussed, as class structures were considered a more accurate framework for understanding French political life. However, the rise of identity-based narratives emphasizing French roots, often with a populist undertone, combined with the influences of European integration and globalization, has brought the content of citizenship back into focus, particularly in its relationship to nationality.

### 4.2 Nationality, a legal status

Nationality is a legal status that is defined by law, whereas citizenship is a philosophical concept rooted in the 1789 Declaration of the Rights of Man and of the Citizen. In France, the definition of nationality represents a compromise between *jus sanguinis* and *jus soli*. During the *Ancien Régime*, nationality was primarily based on *jus soli*: individuals were tied to the land of their lord, and their territorial belonging defined their nationality. This changed with Napoleon Bonaparte, who replaced *jus soli* with the *jus sanguinis* in the Civil Code of 1804. Similar reforms were implemented in other European countries conquered by the Empire. The United Kingdom, which was never invaded by Napoleon, retained its traditional *jus soli* system and later applied it in its settler colonies, including the United States, Canada, Australia, and New Zealand.

At the time, nationality was not a significant concern. Passports, aside from those issued to diplomats, did not exist until the nineteenth century, and identity cards were introduced in France only in 1917, remaining non-mandatory to this day. The French census first differentiated nationals from foreigners in 1851. Prior to this, the Ministry of the Interior primarily identified foreigners as political activists associated with the revolutions of 1830 and 1848, who were tracked by the police.

The demographic decline, which became evident in France earlier than in its neighboring countries, starting at the end of the eighteenth century, raised concerns during the economic boom of the second half of the nineteenth century (Vallin, 2002). After years of debate over “denationalization or depopulation”, the 1889 law introduced a significant reform to the nationality code, incorporating the principle of *jus soli* into the French Civil Code of Napoleon, which had previously been based on *jus sanguinis*. This marked the beginning of a long compromise that eventually led to a balanced approach between the two sources of access to nationality.

The nationality code underwent further reforms in 1927, 1945, and 1973, expanding access to French nationality through the principle of *jus soli*. However, these changes sparked relatively few public political debates. It was only in the 1990s that the question of nationality returned to the forefront, driven by the National Front and the *Club de l'Horloge*, which promoted slogans such as “We must deserve to be French” and “There are people who are French only on paper, despite themselves”. In October 1985, the right-leaning journal Le Figaro Magazine ran a provocative headline: “Will we still be French in 30 years?”

In 1987, the French government appointed a commission to deliberate on the reform of the nationality code. After conducting around 100 hearings, the commission ultimately decided not to amend the 1973 law. At the time, the right-wing favored strengthening *jus sanguinis*, while the left-wing advocated for expanding *jus soli*, emphasizing the development of a sense of citizenship through participation in local life and social integration via residency.

When the right-wing returned to power in 1993, it passed the Pasqua-Méhaignerie law, introducing stricter requirements for acquiring nationality for second-generation migrants born in France to foreign parents. The law eliminated automatic nationality acquisition at age 18 and barred individuals with criminal sentences exceeding 6 months from ever becoming French. It also ended the provision allowing the children of Western Africans from Senegal (Saint-Louis, Rufisque, and Dakar), who were themselves French citizens, to acquire French nationality. Only Algerians could still apply for reintegration into French nationality if their parents or grandparents had acquired full citizenship through civil service or military service. The left-wing and human rights associations strongly opposed this law.

In 1998, when the left-wing regained power, the Guigou law was passed, returning to the principles of the 1973 law. It reinstated automatic French nationality for those born in France who had lived there for at least 5 years before turning 18. This reestablished the balance between *jus sanguinis* and *jus soli*.

Since then, there have been no significant debates on nationality, except for the 2024 Immigration law, inspired by the *Rassemblement National*. However, the proposal to limit access to *jus soli* was struck down by the Constitutional Council.

Certain distinctions have evolved regarding access to civic rights (voting and eligibility) and marriage. The earliest nationality laws established a waiting period during which new citizens were not allowed to vote or run for office (5 and 10 years, respectively), creating a distinction between active and passive citizens. This distinction was abolished by the 1973 law. For marriage, the required length of time to obtain nationality has been extended due to concerns about so-called “white marriages” (marriages of convenience) arranged solely to gain access to French nationality.

### 4.3 Citizenship, an evolutive concept

Nationality and citizenship have not always been closely linked to integration. Assimilation, which dominated in France from the late nineteenth century to the mid-twentieth century as an individual process, has been gradually replaced by integration, which requires less stringent allegiance, and the participation of both the migrant and the country of residence (Lépinard and Simon, 2009). However, neither assimilation nor integration policies have included the granting of local political rights to incorporate foreign citizens in France.

Even though “citizenship of residence” is a slogan supported by many advocacy groups, it remains difficult in France to be a citizen without being a national, except for EU citizens. Nevertheless, the separation of nationality and citizenship has entered political and constitutional debates. Under pressure from Europe, the French Constitution was reformed to comply with the Maastricht Treaty of 1992, which introduced European citizenship, granting local political rights and eligibility to all EU citizens based on reciprocal rights.

Several proposals to grant local political rights to non-EU residents have been introduced, but none have secured a parliamentary majority. While public opinion and the need for constitutional reform are often cited as obstacles, these could potentially be overcome if there were strong political will, something that is currently lacking. The most recent proposal, submitted by the Green, Communist, and Socialist parties in 2000, also failed. Most efforts aimed at promoting social cohesion, such as urban policies, equal opportunity programs, and anti-discrimination measures, do not focus on extending citizens' rights to foreign residents.

Civic identity in France stems from the Revolution, defining the nation as a collective built on shared political values rather than ethnic ties. Born at Valmy in 1792, this idea was later theorized by Ernest Renan, who described the nation as a shared will and collective memory. This republican vision, rooted in inclusivity, was challenged by nationalism, which tied the nation to territory, culture, and even blood, as seen in Maurras' theories. The concept was inconsistently applied, legitimizing colonialism while also fostering exclusion, notably during the Dreyfus affair and under the Vichy regime, when Jews were stripped of citizenship and persecuted.

The rediscovery of citizenship emerged relatively recently, in the mid-1980s. At that time, the political left sought to reclaim the concepts of citizenship and nation from the far right. In response to the right-wing *Club de l'Horloge*, the socialist Club 89 published a book in 1985 emphasizing republican values (*L'identité française*). Immigration began to reshape the definition of citizenship, introducing new values such as social integration through local residence, cultural pluralism, and anti-discrimination efforts, exemplified by the 1983 *Marche des Beurs*.

Islam also brought a new question to the forefront: can one be both French and Muslim? The integration of second and third-generation migrants into French nationality and citizenship saw members of the *Beur* movement voting and running for local office during the municipal elections of 1989 and beyond. These groups formed a political movement through civic associations like SOS Racisme and France Plus, presenting their demands to political parties and positioning themselves as a political force in the 1990s.

Some leaders of this movement actively reinforced their image as exemplary French citizens, embodying all the symbols of the Republic: public schools, secularism, and civic identity, with the sentiment *Plus républicain que moi, tu meurs* (“No one is more republican than me”; Wihtol de Wenden and Leveau, 2001). However, many French citizens did not recognize them as truly French, defining “true French” as those with roots and ancestry tied to France (*Français de souche*). Sociologist Michel Wieviorka describes this phenomenon as “differentialist racism” (Wieviorka, 2004). Those harboring such views were often poor, unemployed, and felt they were in competition with migrants, contributing to the ethnicization of French identity.

In response to discrimination and their “visible” status, some young people from inner cities have a strong sense of attachment to the neighborhood. Many of them identify as dual citizens, even though their countries of origin were initially resistant to this notion. Over time, however, these countries have come to see dual citizenship as a tool for advancing their political and diplomatic interests in France. Countries of origin increasingly encourage quasi-diasporas in immigration destinations, leveraging the multiple allegiances of their compatriots to maintain ties and influence abroad.

Discrimination and terrorism can hinder progress in this area. In France, even though many individuals from second and third generations consider themselves French, a significant portion of the population remains unconvinced of their French identity, creating a divide within the nation. A field study on young people of Arab origin (Brouard and Tiberj, 2005) concluded with the phrase: “French like the others”. Similarly, in another study (Bertossi and Wihtol de Wenden, 2007), one participant, referring to their French colleagues at work, remarked: “They will have made progress when they understand that we are French”.

## 5 Concluding remarks

The aim of this paper was to analyze how migration has influenced the conception of citizenship by challenging the traditional state-citizen relationship. Our research questions were how migration has reshaped the conception of citizenship and what challenges migrants, refugees, stateless individuals, asylum seekers and environmentally displaced persons face in obtaining legal status in host countries. We answered these research questions by reviewing the international scientific literature on the topic.

We found that the concept of citizenship and belonging has become increasingly negotiable, particularly in countries with long histories of immigration and integration. These countries have been compelled to adopt values such as multiculturalism and the fight against discrimination to integrate migrants as future citizens, despite the growing prominence of nativist sentiments, often fueled by populist movements. Many European countries, such as Belgium, France, Portugal and Spain, balanced *jus soli* and *jus sanguinis* principles to facilitate access to citizenship for newcomers and their children born in the host country. Moreover, EU citizenship, which grants Europeans the right to vote and stand for election in the European Parliament as well as in local elections, has further dissociated nationality from citizenship. Brubaker (2000) highlighted the significant influence of nationality laws and naturalization policies on newcomers' political integration. However, there is no consistent trade-off between countries that are more open to nationality and those that provide local political rights to their residents (Yilmaz and Wolffhardt, 2024).

While mobility is often considered a hallmark of modernity, most people from the Global South cannot migrate legally or access lawful residency in the Global North. The contradictions of a world where everything circulates except for human beings has led to the rise of populations without formal status, including rejected asylum seekers, undocumented migrants, stateless individuals, and those displaced by environmental crises. Civil societies in immigration countries have played a crucial role in defending access to rights. Similarly, in countries of origin, the rise of migration diplomacy in international forums has fostered greater inclusion. Additionally, migrant practices, like dual citizenship, transnationalism, and naturalization have provided ways to navigate closed borders and restrictive policies. Migrants have disrupted the classical notion of citizenship, which sees individuals residing exclusively within their states, giving rise to various forms of transnational citizenship.

Finally, the case study of France, illustrative of the evolution of the understanding of citizenship due to international migration, showed that citizenship is based on the principles of the social contract and participation, extended to foreigners and their descendants. In contrast, nationality remains a distinct legal concept. In particular, it is difficult to be a citizen without being a national, except for EU citizens. Neither assimilation nor integration policies have granted local political rights to foreign citizens in France. However, the consolidation of migration flows and the European integration process have introduced new values of belonging, such as diversity, pluralism and anti-discrimination. Nevertheless, the equilibrium between *jus soli* and *jus sanguinis* continues to be challenged by populist movements, which are rather adverse to multiculturalism.
